# PKQuest_Java: free, interactive physiologically based pharmacokinetic software package and tutorial

**DOI:** 10.1186/1756-0500-2-158

**Published:** 2009-08-05

**Authors:** David G Levitt

**Affiliations:** 1 Department of Integrative Biology and Physiology, University of Minnesota, 6-125 Jackson Hall, 321 Church St. S. E., Minneapolis, MN 55455, USA

## Abstract

**Background:**

Physiologically based pharmacokinetics (PBPK) uses a realistic organ model to describe drug kinetics. The blood-tissue exchange of each organ is characterized by its volume, perfusion, metabolism, capillary permeability and blood/tissue partition coefficient. PBPK applications require both sophisticated mathematical modeling software and a reliable complete set of physiological parameters. Currently there are no software packages available that combine ease of use with the versatility that is required of a general PBPK program.

**Findings:**

The program is written in Java and is available for free download at . Included in the download is a detailed tutorial that discusses the pharmacokinetics of 6 solutes (D_2_O, amoxicillin, desflurane, propofol, ethanol and thiopental) illustrated using experimental human pharmacokinetic data. The complete PBPK description for each solute is stored in Excel spreadsheets that are included in the download. The main features of the program are: 1) Intuitive and versatile interactive interface; 2) Absolute and semi-logarithmic graphical output; 3) Pre-programmed optimized human parameter data set (but, arbitrary values can be input); 4) Time dependent changes in the PBPK parameters; 5) Non-linear parameter optimization; 6) Unique approach to determine the oral "first pass metabolism" of non-linear solutes (e.g. ethanol); 7) Pulmonary perfusion/ventilation heterogeneity for volatile solutes; 8) Input and output of Excel spreadsheet data; 9) Antecubital vein sampling.

**Conclusion:**

PKQuest_Java is a free, easy to use, interactive PBPK software routine. The user can either directly use the pre-programmed optimized human or rat data set, or enter an arbitrary data set. It is designed so that drugs that are classified as "extracellular" or "highly fat soluble" do not require information about tissue/blood partition coefficients and can be modeled by a minimum of user input parameters. PKQuest_Java, along with the included tutorial, could be used as the basis of an interactive, on-line, pharmacokinetic course.

## Background

Physiologically Based Pharmacokinetics (PBPK) refers to the approach of modeling drug kinetics using a realistic physiological description of the animal [1,2]. The PBPK modeling approach has remained primarily in the realm of the specialist because of its requirement for both sophisticated mathematical software and a reliable set of physiological parameters describing the model organs. The PBPK software available as of 2002 was reviewed by Rowland et. al. [3]. The software that has been adapted for PBPK modeling varies from general scientific computing packages (e.g. MATLAB) to biological and pharmacokinetic routines (e.g. ADAPT II, NONMEM, WinNonlin, SAAM) to proprietary software designed for specific applications that are difficult to adapt for general use (e.g. GastroPlus, Physiolab). More recently, Barboriak et. al. developed a freely distributed, dedicated PBPK model using JSim [4]. None of these programs provide the combination of an easy to learn, intuitive interface with the wide-ranging features that are necessary for the most general applications.

PKQuest, a freely distributed general purpose PBPK program, was released in 2002 [5]. It has now been used in the analysis of the pharmacokinetics of more then 30 different solutes in a series of publications [5-14]. Using and refining the same parameter set for this large number of disparate solutes provides increased confidence in the validity and generality of the physiological parameters. Because PKQuest was written in Maple (Maplesoft), it required the purchase, installation and some minimal training in Maple. The purpose of this paper is to introduce PKQuest_Java, a completely revised, Java based, interactive version of PKQuest. It is available for free download at . It requires that a recent version of the Java Runtime Environment (JRE) is installed on the computer where it will be run. The main features and a brief tutorial on its use are described below. Since its interactive design is intuitive and there is little installation required, the easiest way to learn about it is to simply download and run it.

PBPK modeling presents an ideal approach to introduce students to the dependence of pharmacokinetics on the interaction between the physical chemical properties of a drug and physiology. Included in the download is a detailed tutorial that takes the student through the pharmacokinetics of a series of 6 solutes in the human (D_2_O, amoxicillin, desflurane, propofol, ethanol and thiopental) and two solutes in the rat (sevoflurane and propofol). These examples use actual experimental data and provide a good introduction to the major features of PKQuest_Java. All of the parameters for each solute are stored in Excel "example" files that are also included in the download. This tutorial could form the basis of an online pharmacokinetics course.

## Implementation

The major features of PKQuest_Java are:

1) Intuitive and versatile interactive interface.

2) Absolute and semi-logarithmic graphical output.

3) Pre-programmed optimized human and rat parameter data set (but, arbitrary values can be input).

4) Time dependent changes in the PBPK parameters.

5) Non-linear parameter optimization.

6) Unique approach to determine the oral "first pass metabolism" of non-linear solutes such as ethanol.

7) Pulmonary perfusion/ventilation heterogeneity for volatile solutes.

8) Input and output of Excel spreadsheet data.

9) Antecubital vein sampling.

**Figure 1:**
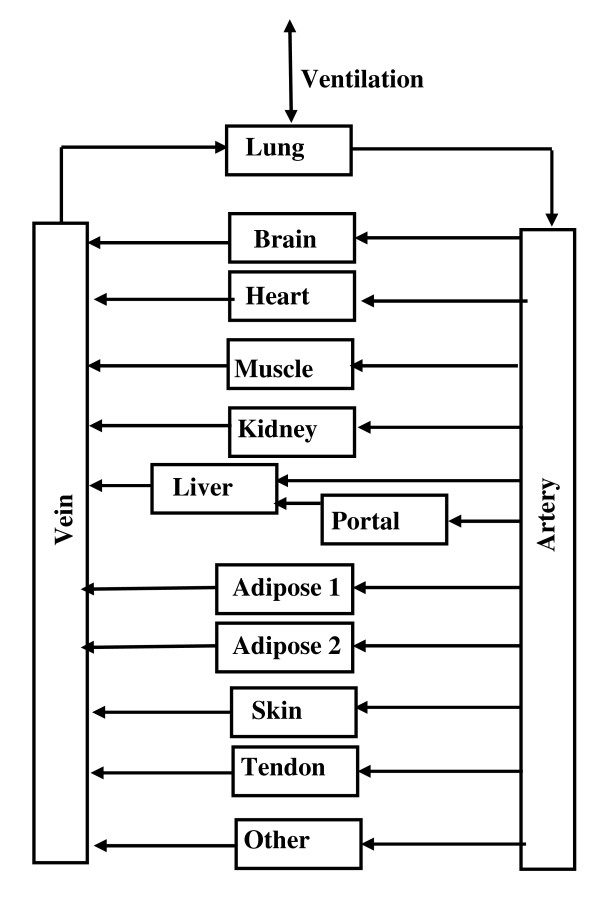
**Flow diagram and arrangement of organs used for the PBPK model**. Each box represents a well-stirred compartment and each arrow is an input or output to the organ. The organ labeled "Portal" includes all organs drained by the portal vein (stomach, small and large intestine, spleen and pancreas). The organ labeled "Other" represents "connective" tissue and its weight is adjusted during the run to so that total weight is equal to that set by the subject's "weight". Adipose tissue is represented by two equal volume adipose compartments with different perfusion rates.

PKQuest_Java uses the standard "whole body" PBPK organ model (Figure 1) with each organ characterized by an adjustable volume, blood flow, capillary permeability, metabolism and tissue/blood partition coefficient. The major factor limiting the utility of the PBPK approach is uncertainty about the value of the tissue/blood partition coefficient for the different organs. Since this partition depends on specific and highly variable drug tissue binding properties, each drug has a unique set of values [15]. However, there are two drug classes for which this partition can be predicted just from in vitro measurements. The first is the class of "extracellular" solutes (e.g. β-lactam antibiotics) for which the partition depends primarily on interstitial albumin binding which can be predicted using in vitro measurement of plasma binding and previous knowledge of tissue albumin concentration [10]. The second class is that of the "highly fat soluble" drugs for which the partition coefficient can be accurately predicted from in vitro measurements of the fat/water partition coefficient and previous determinations of tissue fat content [7,12,13]. PKQuest_Java is designed so that these special cases can be directly selected, allowing prediction of the pharmacokinetics with a minimum of user input.

**Figure 2:**
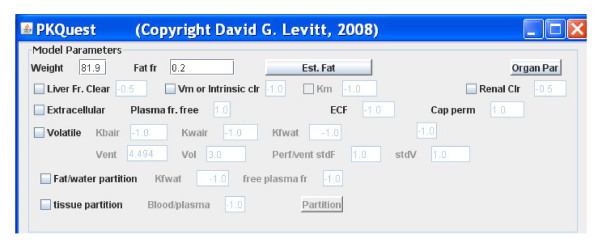
**Snapshot of the "Model Parameters" window that opens when PKQuest_Java is first run**.

**Figure 3:**
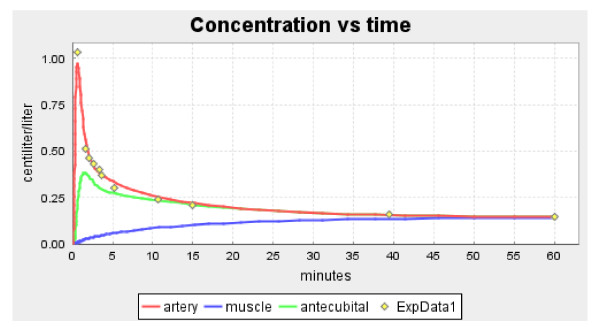
**Snapshot of the "Output" window that is opened when the default PKQuest_Java (D_2_O kinetics) is run**. The concentration versus time of the arterial, muscle and antecubital vein water concentrations are displayed along with the experimental data.

When PKQuest_Java first opens, a series of windows are displayed (Model Parameters, Input, Plot, etc.). The "Model Parameter" window is shown in Figure 2. The default model parameters when the program is run are those used for modeling the pharmacokinetics of D_2_O. This is the simplest possible case, with D_2_O distributing in all the body water and no metabolism or significant excretion during the time of the experiment. The only input required is the D_2_O dosing, which for the default case corresponds to the experimental conditions described by Schloerb et. al. [16]. Clicking the "Run" button (no user input required), the default case is run and the standard "Output" window opens (Figure 3). The concentration versus time plot (either absolute or semi-log) is plotted for all of the organs selected by the user. Three plots are shown in Figure 3: the arterial, muscle and "antecubital" water concentration along with the experimental data. (A unique feature of PKQuest_Java is that an estimate of the antecubital concentration is provided based on the assumed mixing that occurs at this site [11]). PKQuest_Java allows non-linear optimization of all the major parameters. In the tutorial this feature is illustrated by adjusting the muscle blood flow (which dominates the D_2_O kinetics) to optimize the fit to the experimental data. This default case used the set of organ parameters that have been optimized by application of PKQuest to a large number of different solutes and these parameters are automatically scaled for body weight and fat fraction. In general, unless one has good reason to assume that these values are incorrect (e.g. increased muscle blood flow during exercise) they should not be modified. Arbitrary values for the organ perfusion, volume and capillary permeability can be input into a table opened by clicking the "Organ Par" button (Figure 2).

The pharmacokinetics of amoxicillin is discussed in the tutorial as an example of a typical "extracellular" solute for which the tissue/blood partition can be predicted from in vitro measurements of plasma binding. This drug class is selected by clicking the "Extracellular" box which activates 3 other input boxes (see Figure 2): 1) the free plasma fraction, 2) A factor that adjusts the default extracellular volume of each organ; and 3) A capillary permeability factor (= 1.0 for amoxicillin which is flow limited). In addition, a value needs to be entered in the renal clearance box (Figure 3).

Two "high fat soluble" drug classes are discussed in the tutorial as examples of drugs whose tissue/partition coefficient can be predicted from in vitro measurements of fat/water partition. The first are the volatile anesthetics, represented by modeling the experimental desflurane pharmacokinetics [12]. Since the metabolism of most volatile anesthetics is negligible, their elimination rate is completely determined by the physiological ventilation parameters. This class is selected by clicking the "Volatile" check box and entering the values of blood/air, water/air and fat/water partition coefficients (see Figure 2). Optionally, the alveolar volume and ventilation rate can be input if they differ from the default values. A unique feature of PKQuest_Java is that it can be used to model perfusion-ventilation heterogeneity. The boxes labeled "Perf/vent stdF and stdV characterize the values of the perfusion and ventilation heterogeneity (normal value = 1). The long time kinetics of these solutes is dominated by the adipose tissue exchange and an accurate model requires a heterogeneous adipose perfusion [12]. This is modeled in PKQuest_Java by two equal volume adipose compartments with different perfusion rates (see Figure 1). The second "high fat soluble" drug in the tutorial focuses on the pharmacokinetics of the non-volatile propofol [13] and is selected by clicking the Fat/water partition box and filling in the fat/water partition and, optionally, the fraction free in plasma (if it differs from the default value). The propofol experimental data that is modeled has a complex input (2 different constant inputs and 1 exponential input) which is illustrated in the tutorial.

One class where PBPK modeling is a necessity is for drugs with non-linear metabolism. For example, in order to accurately model the non-linear ethanol pharmacokinetics it is necessary to determine the Vmax and Km for the liver metabolism. This requires an accurate simulation of the liver tissue concentration through a modeling of liver perfusion. The tutorial focuses on ethanol and illustrates, through a series of problems, the main pharmacokinetic features of non-linear solutes. A unique feature of PKQuest_Java is that it provides a rigorous definition and procedure for determining "first pass metabolism" using two sets of experimental data: the blood concentration following 1) intravenous and 2) oral input. It uses the following procedure: Firstly, the non-linear PBPK parameters are determined using the IV input data. Secondly, the time course of the intestinal absorption that would produce the observed blood values following an oral input is determined. (The intestinal absorption is modeled in the form of the Hill equation). Finally, the IV input required to fit this same blood concentration data following oral input is determined and the "first pass metabolism" is defined as the difference between the calculated oral and IV inputs. Although this seems to be a complicated procedure, it is made simple and transparent in PKQuest_Java and is discussed in detail in the tutorial.

The above examples all used solutes for which the tissue/blood partition coefficient can be determined from in vitro measurements. Unfortunately, the great majority of solutes are exceptions to this. Although rough estimates (within a factor of about 2) of this partition can be made from drug structure algorithms, accurate estimates usually require direct animal measurement of tissue/plasma partition [15]. This general partition case is illustrated in the tutorial using thiopental. It is selected by clicking the "tissue partition" check box and then clicking the "Partition" button (see Figure 2) which opens a table that requires the user to fill in the values of the tissue/plasma partition for each organ in the table.

The most important application of PBPK modeling is in the area of toxicokinetics and environmental risk assessment where it is essential to simulate local tissue concentrations under varying exposure conditions (e.g. rest, exercise, etc.) [17,18]. For this analysis, the PBPK model must be able to simulate time dependent changes in the physiological parameters (e.g. ventilation rate, cardiac output, muscle blood flow). The design of PKQuest_Java provides a relatively simple procedure to model step changes in the PBPK parameters. One first develops (and saves in Excel files) separate models for each set of the constant PBPK parameters. Then, using an interactive option, one lists the order that the individual models are called and the time point when the switch is made and then the complete sequential set is run. This procedure is illustrated in the tutorial by the modeling of the experimental long time (6 day) washout of desflurane during a period when the subject's alveolar ventilation varied as the conditions varied from anesthesia to ambulatory [12].

## Conclusion

PBPK modeling still remains a subject for specialists, primarily in the area of environmental risk assessment. Highly refined sophisticated software that satisfies regulatory requirements has been developed to meet these needs. PKQuest_Java is not designed as a substitute for these programs. In addition, PKQuest_Java makes no attempt to incorporate population modeling which adds another level of sophistication and training and is superbly described by NONMEM. PKQuest_Java is designed for the non-specialist who would like to attempt some PBPK modeling without acquiring detailed training or expensive software. In addition, it is hoped that PKQuest_Java along with the included tutorial can be used as an introduction to pharmacokinetics for students (possibly as an on-line tutorial). The version now being distributed is a first attempt and the author welcomes suggestions for modifying it to add additional features, make it more user friendly or increase its value as a student learning tool.

## Availability and requirements

The jar file, tutorial and example files are now available for download from 

PKQuest_Java requires that a recent version of the Java Runtime Environment (JRE) is installed on the computer where it will be run.

The software application is freely available to anyone wishing to use it for non-commercial purposes, without restrictions such as the need for a material transfer agreement.

## List of abbreviations used

PBPK: Physiologically based pharmacokinetic modeling.

## Competing interests

The author declares that they have no competing interests.

## Authors' contributions

DGL developed the software, the examples and the tutorial and has read and approved the final manuscript.

## References

[B1] Reddy MB, Yang RSH, Clewell HJ, Andersen ME (2005). Physiologically Based Pharmacokinetic Modeling.

[B2] Edginton AN, Theil FP, Schmitt W, Willmann S (2008). Whole body physiologically-based pharmacokinetic models: their use in clinical drug development. Expert Opin Drug Metab Toxicol.

[B3] Rowland M, Balant L, Peck C (2004). Physiologically based pharmacokinetics in drug development and regulatory science: a workshop report (Georgetown University, Washington, DC, May 29-30, 2002). AAPS PharmSci.

[B4] Barboriak DP, MacFall JR, Viglianti BL, Dewhirst Dvm MW (2008). Comparison of three physiologically-based pharmacokinetic models for the prediction of contrast agent distribution measured by dynamic MR imaging. J Magn Reson Imaging.

[B5] Levitt DG (2002). PKQuest: a general physiologically based pharmacokinetic model. Introduction and application to propranolol. BMC Clin Pharmacol.

[B6] Levitt DG (2002). PKQuest: capillary permeability limitation and plasma protein binding - application to human inulin, dicloxacillin and ceftriaxone pharmacokinetics. BMC Clin Pharmacol.

[B7] Levitt DG (2002). PKQuest: volatile solutes - application to enflurane, nitrous oxide, halothane, methoxyflurane and toluene pharmacokinetics. BMC Anesthesiol.

[B8] Levitt DG (2002). PKQuest: measurement of intestinal absorption and first pass metabolism - application to human ethanol pharmacokinetics. BMC Clin Pharmacol.

[B9] Levitt DG (2003). The use of a physiologically based pharmacokinetic model to evaluate deconvolution measurements of systemic absorption. BMC Clin Pharmacol.

[B10] Levitt DG (2003). The pharmacokinetics of the interstitial space in humans. BMC Clin Pharmacol.

[B11] Levitt DG (2004). Physiologically based pharmacokinetic modeling of arterial - antecubital vein concentration difference. BMC Clin Pharmacol.

[B12] Levitt DG (2007). Heterogeneity of human adipose blood flow. BMC Clin Pharmacol.

[B13] Levitt DG, Schnider TW (2005). Human physiologically based pharmacokinetic model for propofol. BMC Anesthesiol.

[B14] Levitt DG, Schoemaker RC (2006). Human physiologically based pharmacokinetic model for ACE inhibitors: ramipril and ramiprilat. BMC Clin Pharmacol.

[B15] Fagerholm U (2007). Prediction of human pharmacokinetics - evaluation of methods for prediction of volume of distribution. J Pharm Pharmacol.

[B16] Schloerb PR, Friis-Hansen BJ, Edelman IS, Solomon AK, Moore FD (1950). The measurement of total body water in the human subject by deuterium oxide dilution. J Clin Invest.

[B17] Clewell Iii HJ, Andersen ME, Blaauboer BJ (2008). On the incorporation of chemical-specific information in risk assessment. Toxicology Letters.

[B18] Thompson CM, Sonawane B, Barton HA, DeWoskin RS, Lipscomb JC, Schlosser P, Chiu WA, Krishnan K (2008). Approaches for Applications of Physiologically Based Pharmacokinetic Models in Risk Assessment. Taylor & Francis.

